# Preparation duration shapes the goal-directed tuning of stretch reflex responses

**DOI:** 10.1007/s00221-025-07139-z

**Published:** 2025-08-18

**Authors:** Robin Rohlén, Frida Torell, Michael Dimitriou

**Affiliations:** 1 Department of Medical and Translational Biology, Umeå University, Umeå, Sweden; 2 Department of Diagnostics and Intervention, Umeå University, Umeå, Sweden

**Keywords:** Preparatory delay, Reaching task, Stretch reflex, Perturbation, Electromyography

## Abstract

Stretch reflex responses counteract sudden perturbations, and modulation of reflex gains can facilitate voluntary movement. Recent studies suggest movement preparation includes goal-directed tuning of muscle spindles and an equivalent modulation of both short- and long-latency stretch reflex responses (SLR and LLR), as long as the preparatory delay between ‘Cue’ and ‘Go’ exceeds 250 ms. The current study aimed to clarify the minimal preparation time required for goal-directed modulation of SLR and LLR responses and to determine how such modulation progressively evolves with extended preparation. We recorded bipolar electromyographic signals of healthy participants to assess reflex responses to mechanical perturbations induced by a robotic manipulandum in the context of a delayed-reach task. Specifically, we examined how multiple preparatory delays (250, 300, 350, 400, 450, and 500 ms) impact the goal-directed modulation of SLR and LLR responses from the loaded or unloaded pectoralis major, anterior deltoid, and posterior deltoid muscles. We found that preparatory delays of 300 ms and 350 ms are sufficient for goal-directed tuning of SLR responses in the posterior deltoid and pectoralis muscles, respectively. Our results also suggest that unloading (i.e., antagonist loading) may facilitate both the earlier emergence and more robust expression of goal-directed SLR tuning. Goal-directed tuning of LLR responses emerged as early as 250 ms of preparation, and such tuning was robust against muscle load conditions, in line with previous findings. We observed no consistent increase in SLR tuning at preparation delays that extended beyond the required minimum, whereas such enhancement was observed at the LLR epoch. These findings clarify the temporal characteristics of goal-directed stretch reflex gains, which likely emerge through the interplay of multiple feedback mechanisms.

## Introduction

Voluntary movements are typically preceded by a preparatory phase in which neural signals are modulated in anticipation of motor execution (Kutas and Donchin 1974; Wise 1985; Ghez et al. 1997). During this preparatory period, neural activity has been shown to predict several movement parameters, such as direction (Tanji and Evarts 1976), velocity (Churchland et al. 2006), extent of reach (Messier and Kalaska 2000), trajectory (Hocherman and Wise 1991), and the spatial location of the visual target (Batista et al. 2007). This phase of preparation also includes the formulation of anticipatory motor commands and the tuning of feedback systems that influence long-latency stretch reflex responses (LLRs) before any physical action begins (Todorov and Jordan 2002; Shadmehr and Krakauer 2008; Wagner and Smith 2008; Ahmadi-Pajouh et al. 2012; Yeo et al. 2016).

When an unexpected stretch occurs in the upper limb muscles, it can elicit a short-latency reflex (SLR) within approximately 25 ms and a subsequent LLR around 50 ms post-perturbation onset (Hammond 1956; Marsden et al. 1972). These reflex responses are generally considered involuntary or at least under subconscious or ‘implicit’ control, with volitional control of movement emerging around 100 ms after the initial sensory input (Yang et al. 2011). Traditionally, stretch reflexes have been understood as mechanisms that contribute to the maintenance of posture by counteracting unanticipated disturbances (Nichols and Houk 1976). However, it is now recognised that reflex responses can also be implicitly modulated in a goal-dependent manner in the context of voluntary action. For example, reflex modulation has been observed in response to variations in target features (Nashed et al. 2012), the presence of obstacles (Nashed et al. 2014), and dynamic or static target properties (Cluff and Scott 2015), as well as based on directional cues (Pruszynski et al. 2008; Scott 2016).

At the level of the upper limb, the SLR typically exhibits a dependency on initial muscle load – an effect known as automatic gain scaling – while the LLR demonstrates more nuanced task-specific tuning (Pruszynski et al. 2009; Pruszynski et al. 2011). Recent evidence further differentiates the early and late components of the LLR: the early phase appears to include a stabilising element that operates independently of the voluntary action being prepared, while the later phase reflects task-specific adaptation (Lee and Perreault 2019). These insights support the idea that components of the LLR can be flexibly modulated and may not be purely reflexive in nature (Shemmell et al. 2010). As previously described, feedback mechanisms responsible for modulating LLRs are believed to be configured during the preparatory stage of reaching (Ahmadi-Pajouh et al. 2012). Although the precise mechanisms linking preparatory neural activity to motor output remain elusive, current theories suggest that preparatory activity helps establish the initial neural state necessary for executing goal-directed movement (Churchland et al. 2010; Churchland et al. 2012).

Recent evidence suggests that tuning of muscle spindle sensitivity represents an independent component of movement preparation, contributing to the modulation of reflex gains in a task-relevant manner (Papaioannou and Dimitriou 2021). That is, unlike classic ‘alpha-models’ that subsume all action under a single cortical motor command, recent studies support the presence of an independent fusimotor channel for goal-directed reflex tuning (Dimitriou 2021, 2022). By modulating the responsiveness of spindles and associated reflexes via gamma control, the nervous system can influence sensorimotor feedback independently of any musculoskeletal forces generated during preparation. For instance, the alignment of reflex gain modulation with target direction facilitates more efficient movement execution (Torell et al. 2023). This tuning of stretch reflex gains – observed even at the SLR level – was most evident when the involved muscle was unloaded and when a preparatory delay of 750 ms was present, with a preparation of 250 ms evidently being too short. However, the minimum time required for such tuning to emerge and how this tuning evolves with extended preparation remain open questions.

The overall purpose of this study was to gain further insight into the neural mechanisms that shape stretch reflex responses. Our primary aim was to examine how a series of multiple preparatory delays impacts the goal-directed modulation of stretch reflex gains of the pectoralis major, anterior deltoid, and posterior deltoid muscles. We hypothesised that goal-directed tuning of stretch reflexes would become more intense or likely to emerge following a certain minimum preparation duration. Our experimental design also allowed us to assess the impact of muscle pre-loading or unloading (i.e., antagonist loading) on reflex responses induced at variable preparatory delays. We used electromyographic (EMG) signals to quantify stretch reflex responses of healthy subjects that experienced perturbations of their right upper limb introduced by a robotic manipulandum during a delayed-reach task. Our findings clarify the minimal preparatory time needed for goal-directed tuning of stretch reflex gains and how such gains relate to muscle loading and extended preparation.

## Methods

### Subjects

A total of 21 right-handed and neurologically healthy subjects (10 males of mean age 27.1 ± 5.3 years and 11 females of mean age 24.1 ± 2.1 years) participated in the study. The sample size was selected based on previous studies with a similar scope and experimental setup, where such studies included from 8 to 20 human subjects (Pruszynski et al. 2009; Yang et al. 2011; Dimitriou et al. 2012; Weiler et al. 2021; Torell et al. 2023). Data from five subjects were not included in statistical analyses, one due to poor task performance (participant did not adhere to instructions), one due to a relatively high proportion of trials with variable baseline kinematics during movement preparation (see below), and three due to low-quality EMG signals. The remaining 16 subjects were eight males of mean age 27.6 ± 5.9 years and eight females of mean age 24.8 ± 1.9 years. All 21 subjects were financially compensated for their participation (400 SEK, which is about 40 USD) and gave their informed, written consent before the experimental session commenced. This study was performed in line with the principles of the Declaration of Helsinki and was approved by the Regional Ethical Review Board in Umeå.

### Experimental setup

**Fig. 1:**
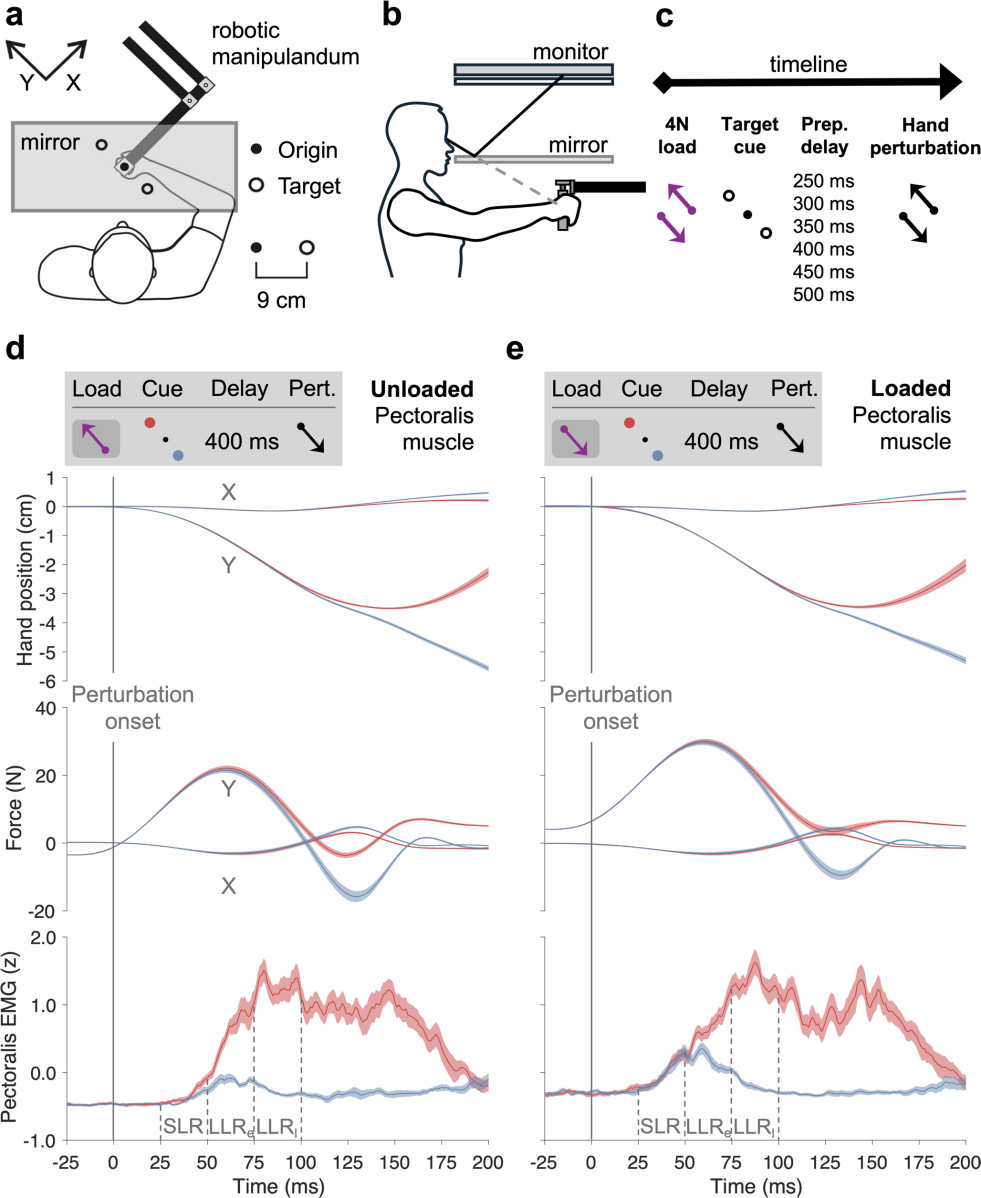
The experimental setup and exemplary data. **a**–**b** Subjects were seated upright in a custom-designed adjustable chair with their right dominant hand secured to a handle. The hand’s position was represented by a visual cursor. All visual stimuli (i.e., cursor, origin and targets) were projected onto a one-way mirror, preventing the subjects from seeing their hand (for details, see Sect. “Methods”). **c** Each trial began when the cursor was placed in the origin. A relatively slow-rising mechanical load was then applied in the front-and-left and right-and-back direction (135 and 315 degrees, respectively). During this phase of the task, participants had to resist the load and maintain their hand at the origin. A visual target was then cued (through becoming a red-filled circle) for 250, 300, 350, 400, 450 or 500 ms. A position-controlled perturbation of the hand was then applied in the 135- or 315-degree direction. The visual cursor’s position was frozen at the origin during the mechanical perturbation, but was otherwise aligned with hand position. After the perturbation, the cued target turned into a green-filled circle corresponding to a “Go” signal, and the subjects had to complete the reaching movement, i.e., move their hand so that the cursor hits the target. **d**–**e** Exemplary data across subjects, i.e., the median hand positions, forces, and z-scored EMG activity across subjects for trials with a preparatory delay of 400 ms when the pectoralis was unloaded (**d**) or loaded (**e**), followed by a perturbation (black arrow) in the direction of pectoralis stretch. For illustration purposes, the visual targets are represented as red and blue, although all targets were red when cued and became green to indicate ‘Go’ in the experimental sessions (see Sect. “Methods”). The vertical dashed lines indicate the epochs of the short- and long-latency stretch reflexes (i.e., SLR, early LLR – ‘LLR_e_’, and late LLR – ‘LLR_l_’). Curve shading denotes ± 1 SE. To simplify display and analyses, we rotated the 2D axes 45 degrees counterclockwise, such that the ‘Y’ axis aligned with the perturbation and visual target axis (see also axes in ‘**a**’)

Subjects were seated upright in a custom-designed adjustable chair positioned in front of the Kinarm End-Point Robot (BKIN Technologies). Using their right hand, they held onto the robotic manipulandum (Fig. 1a–b). The right forearm was supported within a custom foam structure, which rested atop an airsled system that permitted nearly frictionless movement within a two-dimensional workspace. To establish a stable mechanical linkage, the forearm, foam-mounted airsled, manipulandum handle, and hand were all secured together using a Velcro-fastened leather strap. This setup also ensured that the wrist remained aligned with the forearm in a neutral position throughout the experimental session. The robotic platform measured kinematic data regarding the hand’s position, and sensors inside the robotic handle recorded the forces exerted by the subjects’ right hand (six-axis force transducer; Mini40-R, ATI Industrial Automation).

Bipolar surface EMG electrodes (Bagnoli DE-2.1, Delsys Inc., Natick, MA, USA) with 10.0 × 1.0 mm silver contacts and 10 mm inter-electrode distance were placed near the muscle bellies of the pectoralis major, posterior deltoid, and anterior deltoid muscles along the direction of the muscle fibres. We selected these muscles because of their previously demonstrated preferential engagement in a similar task involving the same perturbation and loading directions at the employed workspace (Torell et al. 2024). Before placing the electrodes, the skin was shaved, if necessary, and cleaned with alcohol. A double-sided adhesive tape was then attached to the electrodes and coated with conductive gel. The electrodes were secured using surgical tape to ensure good contact throughout the session. A ground electrode (Dermatrode HE-R Reference Electrode type 00200–3400, 5 cm diameter, American Imex) was placed on the processus spinosus of the C7 region. The EMG signals were sampled at 1 kHz, bandpass filtered at 20–500 Hz, and A/D converted with 16-bit resolution using the Bagnoli EMG amplifier (Delsys Inc., Natick, MA, USA). Position, force, and EMG data were recorded at a sampling rate of 1 kHz using the Kinarm Dexterit-E 3.9.2 software.

### Experimental design and protocol

The experimental setup and the delayed-reach task involving mechanical perturbations are summarised in Fig. 1a-c and are similar to those used elsewhere, e.g., (Torell et al. 2023). Specifically, a one-way mirror prevented the view of the hand and robotic handle, and visual stimuli were projected in the plane of movement. Visual stimuli included a moving white cursor of 1 cm in diameter, representing the hand’s position. In addition, two targets (1.25 cm radius) located 9 cm from the centre point (‘origin’; 0.75 cm radius) were displayed as orange circle outlines unless cued. The targets were placed in a front-and-left and right-and-back direction, corresponding to 135 and 315 degrees.

Each trial started when the cursor was placed inside the origin circle through equivalent movement of the robotic handle by the subject’s right hand. The cursor had to remain steady inside the origin for a randomly generated time (1–1.5 s), after which a 4N load with an 800 ms rise time and 1200 ms hold time was applied by the robotic manipulandum in the 135- or 315-degree direction (Fig. 1c). The subjects were instructed to counter this load so that the cursor remained immobile within the origin. Then, while the load was maintained, one of the two targets turned into a filled red circle (‘Cue’-signal). After a pre-defined but randomly selected preparatory delay (i.e., either 250, 300, 350, 400, 450 or 500 ms), the cued target suddenly turned green (the ‘Go’-signal) and a mechanical perturbation of the hand was applied in one of the two directions (3.5 cm with 150 ms rise-time and no hold). The position-controlled perturbation was designed to approximate a bell-shaped velocity curve, inducing the kinematics of a fast, naturalistic reaching movement (Dimitriou 2018); see also the kinematic signals of Fig. 1d–e. During the perturbation, the position of the visual cursor was frozen at the origin, to prevent the generation of any visuomotor feedback responses. Upon completion of the perturbation, the cursor again resumed to reflect the veridical position of the handle.

At the end of the perturbation, when control of the handle was suddenly returned to the subjects, they had to complete the reaching movement to the cued target, i.e., take the visual cursor to the green cue, where it had to remain immobile for 300 ms. A correct trial was defined as reaching the target between 400 and 1400 ms after perturbation onset. At the end of the trial, the subject received visual feedback on their performance. The subsequent trial began after the cursor was returned to the origin. Because our task employed randomisation of both perturbation direction and pre-load, and because reach preparation in delayed-reach tasks does not induce any overtly detectable change in alpha-drive, any systematic goal-directed intrinsic or ‘pre-flex’ contribution to the SLR or LLR is precluded. Pre-flexes (Brown and Loeb 2000) are critically important in static postural control but are not recruited under these conditions.

The choice of preparatory delays in the current study was guided by previous findings and current aims. Specifically, in previous studies, it has been demonstrated that a preparatory delay of 250 ms is too short to allow preparatory goal-directed tuning of both SLR and LLR responses, whereas 750 ms is long enough for this purpose e.g., (Torell et al. 2023). Through preliminary pilot studies, a preparatory time of 500 ms was identified as sufficiently long and accommodating a reasonable number of incremental preparatory periods between itself and the shortest delay of 250 ms, without the experiment becoming too lengthy and fatiguing for subjects.

This study represents a full factorial design without centre points, i.e., each block contained 48 unique trials in a randomised order comprising two targets to be cued, two load directions, two perturbation directions, and six preparatory delays (2 × 2 × 2 × 6 = 48 trials). Each block was approximately six minutes long, and the entire session lasted around 1.5 to 2 h, including the duration of breaks for rest. The subjects were recommended to take one-minute breaks after two or three consecutive blocks of trials. However, the subjects were free to rest after completing any trial by pulling the cursor (handle) to the side of the workspace. For the first five subjects, there was a total of 13 blocks of trials, while for the latter subjects, there were 18 blocks, resulting in a total of 864 trials. The number of trials was increased for the latter group to facilitate signal detection via averaging, due to the noisy character of the EMG signals and the presence of ‘false-start’ trials (see Sect. “Results”), which prove to be more frequent and persistent compared to similar studies when only two preparatory delays were used (Papaioannou and Dimitriou 2021). When a false start occurred, no perturbation was imposed, and the subject had to restart the same trial. Since this could have provided information on the length of the preparatory delay in that trial, false-start trials were removed from the data analysis to maintain unpredictability and the overall character of the experimental manipulation, i.e., trials without replacement. Moreover, the trial increase in the latter group was expected to primarily benefit the detection of the relatively small SLR response, which represents an unfavourable signal-to-noise ratio compared to LLR. For transparency, the two aforementioned participant groups are colour-coded differently in the Figs. 4, 5, 6, which, among other things, indicates that the data from the two groups are intermingled.

### Data processing

EMG signals were high-pass filtered using a third-order, zero-phase-lag Butterworth filter with a cutoff frequency of 20 Hz and then rectified. For each trial, the time between issuing the perturbation and movement onset (defined as 5% of peak hand velocity) was virtually identical across trials and subjects, given the position-controlled nature of the hand perturbation (i.e., 18 ms). Therefore, all data were aligned on perturbation onset as determined by the Kinarm and then shifted by a fixed delay of 18 ms. The raw EMG data were normalised by z-scoring, as described elsewhere (Dimitriou 2014, 2016), to allow for inter-subject analysis. Briefly, this normalisation procedure involves concatenating all EMG signals from all blocks and calculating each muscle’s sample mean and standard deviation separately. To simplify display and analyses, the 2D position and force data were rotated 45 degrees counterclockwise, so that the ‘Y’ axis was aligned with the perturbation and target axis (see also Fig. 1).

The first four blocks of trials were viewed as familiarisation trials and were not included in the subsequent analyses (see Sect. “Results”). We also removed a relatively small number of trials (on average 7% across all participants) where the kinematics had a variable baseline when required to be stable, e.g., due to the subject’s shoulders being slightly raised at times, which made them slightly more tense, potentially leading to co-contractions. If the experimenter observed this “tightness”, the subject was asked to drop their shoulders to their more natural, otherwise relaxed position. For the kinematics, the hand velocities (both x- and y-axis) were defined empirically as outliers if they were 15 mm/s from the zero-baseline within the first 15 ms after the perturbation.

Because this study focused on stretch reflex responses, we only analysed data from stretching muscles by concentrating on specific combinations of muscle type and perturbation direction. To simplify analyses of individual muscles, the median EMG signal of each muscle was computed for each subject and trial type (i.e., for each load, perturbation, preparatory delay, and target direction) (Torell et al. 2023). The motivation for using the median is that EMG signals can vary from trial to trial for the same condition, e.g., due to the task not being completely restricted in range of motion, and possibly due to the dynamics of the underlying neural mechanisms. Additionally, bipolar EMG signals are noisy, with some responses being larger than others, resulting in a fat-tailed and possibly skewed distribution, where the median is less adversely impacted than the mean as a representative average. For each subject, the median EMG values corresponding to trials where the cued target was associated with homonymous muscle stretch were subtracted from those where reaching the cued target required homonymous muscle shortening, to produce what is referred to below as goal-directed difference values. As in previous studies, these difference values, otherwise corresponding to equivalent experimental conditions, are taken to represent a measure of goal-directed tuning where the short-, early and late long-latency stretch reflexes (SLR, LLR_e_, and LLR_l_) were computed as averages across the epochs represented in Fig. 1d (25–50 ms, 50–75 ms, and 75–100 ms). Finally, our EMG signals were smoothed with a moving mean of five ms for visualisation purposes only.

### Statistical analysis

Statistical analyses were performed on goal-directed difference values across the SLR, early LLR (‘LLR_e_’), and late LLR (‘LLR_l_’) epochs, as represented e.g., in Fig. 1d. Normality tests were performed using the Shapiro–Wilk test, which consistently rejected the null hypothesis (e.g., see the fat tails in Figs. 4, 5, 6). Therefore, to address whether goal-directed tuning of reflex responses occurs at each preparatory delay and load condition, we employed the Wilcoxon signed‐rank test to goal-directed difference values (see Sect. “Data processing”), in order to assess whether the median deviation from zero reaches statistical significance, reporting alongside each the rank-biserial correlation (r_rb_) as a metric of effect size (according to Cohen’s conventions, values of 0.1/0.3/0.5 correspond to small/medium/large effects). The *p*-values generated by the Wilcoxon tests were subjected to the Benjamini–Hochberg adjustment to account for multiple comparisons across all preparatory delays and both load conditions, separately for each reflex epoch and muscle type. Finally, to examine any within-load differences in goal-directed values across the different delay conditions, we ran separate Friedman repeated-measures tests (the non-parametric analogue of one-way repeated-measures ANOVA) on each 5-delay subset (i.e., excluding the 250 ms delay) and followed up with Dunn–Šidák–adjusted post-hoc pairwise contrasts, where applicable. Recognising from prior work that preparatory delays of 250 ms are insufficient for goal-directed tuning (see also above), SLR data pertaining to the 250-ms delay were not included in the Friedman test, to avoid superfluous findings. For consistency, data pertaining to the 250-ms delay were also excluded from the Friedman test investigating LLR responses. In the presence of a statistical effect in one condition (e.g., unloading) and the absence of a statistical effect in another (e.g., loading), we tested (Keysers et al. 2020) whether the goal-directed tuning of the former condition is larger than that of the latter condition using the Wilcoxon signed-rank test as above, with the same *p*-value adjustment for multiple comparisons. Overall, we used a significance level of 0.05 as the base.

Additionally, we examined the onset of goal-directed tuning in reflex responses using a receiver operating characteristic (ROC) technique (Corneil et al. 2004; Pruszynski et al. 2008). The EMG signals of trials with targets in the direction of homonymous muscle stretch were contrasted to those where the cued target was in the direction of muscle shortening. Given these signals, a ROC curve was generated for each sample (corresponding to 1 ms), representing the probability that an ideal observer could discriminate the target position based on the EMG signals. A discrimination time point was defined as significant when the area under the curve (AUC) of the ROC was defined as larger than or equal to 0.75 for five consecutive samples (Corneil et al. 2004; Pruszynski et al. 2008). We also computed the ‘dog leg time point’, i.e., when the ROC curve deviated from chance (probability of 0.5) (Pruszynski et al. 2008). The ‘dog leg fit’ (a horizontal line around the 0.5-probability and a line deviating towards the discrimination time point at a certain point) was computed based on multiple linear regressions with the AUC values located 25 ms around the discrimination point and the corresponding time samples as the independent variable. The best fit was defined as the one with minimal error, i.e., the one having the smallest sum of squared residuals and squared AUC values of the ‘dog leg baseline’ (after being subtracted by 0.5).

All signal processing and statistical tests were performed in MATLAB (version 2023b; MathWorks).

## Results

### Task performance

**Fig. 2:**
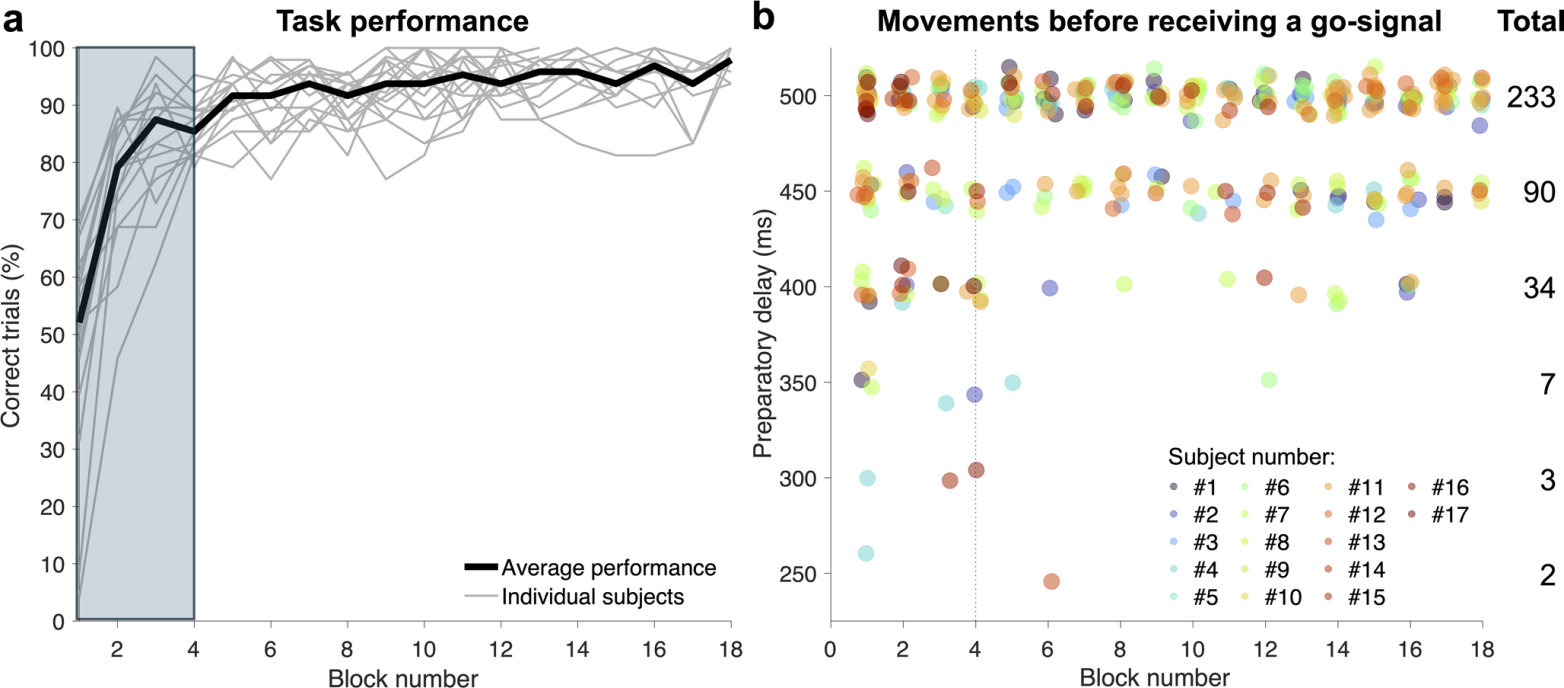
Reach performance and premature movement initiation. **a** The subjects’ performance at the task, in terms of % correct trials in each block of trials. A correct trial involved reaching the cued target within a certain time interval (see main text for more details). Performance reached a high and relatively stable plateau after the first four blocks, which were deemed familiarisation blocks and not included in subsequent analyses (grey background rectangle). **b** Each subject occasionally initiated movements prematurely, i.e., before the ‘Go’ cue (2.6 ± 2.3% of all trials), mainly during the longest preparatory delays. These “false starts” occurred even though the subjects were repeatedly instructed to wait for the ‘Go’ cue. The vertical dotted line represents the final block of familiarisation

The subjects’ performance in the delayed-reach task reached a plateau relatively quickly, with the average performance in terms of % correct trials being above 90% after the first four blocks (Fig. 2a). Therefore, the first four blocks of trials were deemed familiarisation blocks and were not included in subsequent statistical analyses. Although the subjects generally performed well at the task, each subject occasionally initiated movements before receiving the ‘Go’ cue (2.6 ± 2.3% of all trials), a phenomenon mainly observed during the longest preparatory delays, i.e., 450–500 ms with a total of 90 and 233 ‘false starts’, respectively (Fig. 2b). For each participant and each preparatory delay, there were two targets, two loads and two perturbations repeated for 13 or 18 blocks (a total of 2 × 2 × 2 × 13 = 104 or 2 × 2 × 2 × 18 = 144 trials). Summing across all subjects, there was a total of 2104 trials for each preparatory delay. Thus, there were, on average, 4% and 11% false starts at the 450- and 500-ms preparatory delays. For the shorter preparatory delays, most false starts occurred during the familiarisation blocks (Fig. 2b). Although infrequent overall, these false starts persisted despite repeated instructions to wait for the ‘Go’ cue. That is, the experimenter provided relevant instructions before data collection began and repeatedly after false starts, but interestingly, a relatively small number of such trials often persisted throughout the experiment. ‘False-start’ trials were removed from further analyses (see “Methods” for more details).

### Goal-directed modulation of stretch reflex responses

**Fig. 3:**
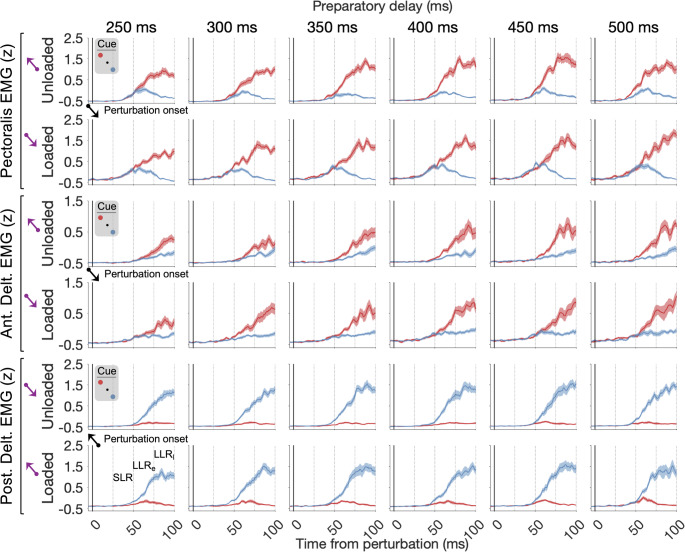
The median z-scored EMG activity across subjects (n = 16) for the unloaded and loaded pectoralis major, anterior deltoid, and posterior deltoid muscles. The conditions are specified by the cued targets (red and blue lines/dots), the load direction before perturbation (purple arrow), and the kinematic perturbation direction (black arrow). Throughout this figure, the data represent trials where the hand was perturbed along the direction of homonymous muscle stretch, whereas “loaded” and “unloaded” refer to the direction of muscle loading that was applied before the kinematic perturbation. The black vertical line at time ‘0’ represents perturbation onset. Curve shading denotes ± 1 SE

All data in the following results pertain to stretch reflex responses, i.e., we focused on trials where the hand was perturbed in the direction of homonymous muscle stretch. The median z-scored EMG activity (across subjects) for the unloaded and loaded pectoralis major, anterior deltoid, and posterior deltoid muscles for all preparatory delays is shown in Fig. 3. As indicated in this figure, goal-directed EMG activity, i.e., the difference between the red and blue curves for the unloaded (loaded) traces, seemed to vary overall as a function of preparatory delay.

### Goal-directed modulation of stretch reflex responses—pectoralis major

A representative example of hand positions, forces, and median z-scored pectoralis EMG activity (across subjects) with a 400 ms preparatory delay is also illustrated in Fig. 1d–e: a preparatory delay of 400 ms appears sufficient for inducing goal-directed tuning of the SLR response when the homonymous muscle is unloaded, i.e., reflected by the differentiation of the EMG curves (red vs. blue) within the SLR epoch when the pectoralis is unloaded. On the other hand, there is goal-directed tuning of the long-latency stretch reflexes (LLR_e_ and LLR_l_) regardless of background load condition.

**Fig. 4:**
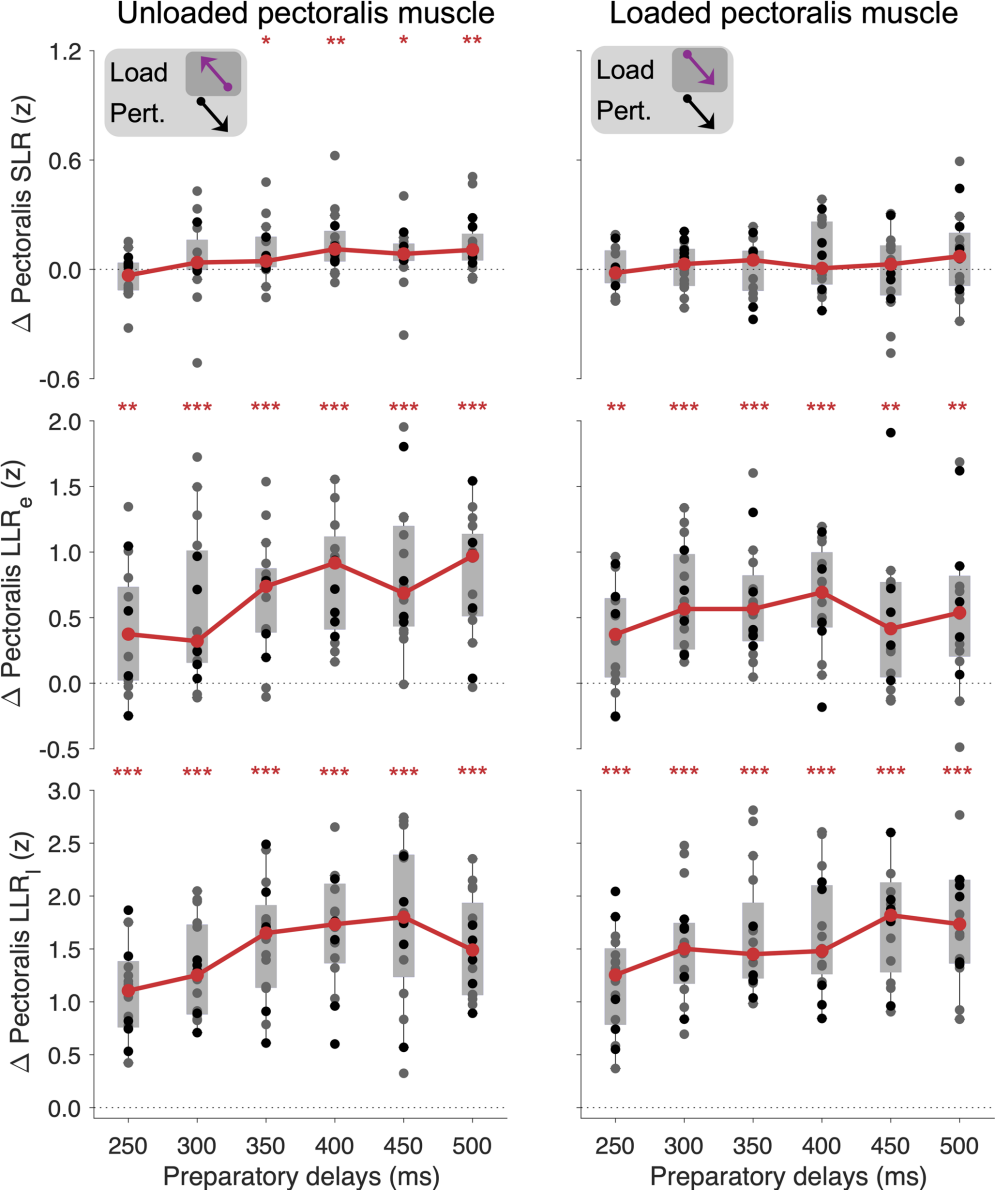
Goal-directed differences in pectoralis EMG for the SLR, LLR_e_, and LLR_l_ epochs. Grey circles represent the median single-subject EMG values of the group performing 18 blocks of trials, whereas the black circles represent the values of the group performing 13 blocks. Grey background rectangles represent upper and lower quartiles, and thin vertical lines represent the 95% data range. Solid red lines represent the group-level median values. *p < 0.05, **p < 0.01, ***p < 0.001 denote significant difference from zero

For the unloaded pectoralis SLR, Wilcoxon tests indicated significant goal-directed tuning (i.e., goal-directed difference values > 0) at all preparatory delays except 250 and 300 ms (Fig. 4, top; N = 16 subjects; 250 ms: W = 47, *p* = 0.511, r_rb_ =  − 0.31; 300 ms: W = 98, *p* = 0.311, r_rb_ = 0.44; 350 ms: W = 115, *p* = 0.039, r_rb_ = 0.69; 400 ms: W = 127, *p* = 0.006, r_rb_ = 0.87; 450 ms: W = 116, *p* = 0.039, r_rb_ = 0.71; 500 ms: W = 129, *p* = 0.006, r_rb_ = 0.90). A Friedman test indicated no significant differences in goal-directed SLR responses among the preparatory delays of 300–500 ms (χ^2^_4_ = 14.6, *p* = 0.211). In accordance with previous findings, Wilcoxon tests indicated no significant goal-directed tuning of the SLR when the pectoralis muscle was loaded, at all preparatory delays (all *p* > 0.462). A direct comparison of pectoralis SLR tuning magnitudes between the unloaded and loaded conditions for (350 to 500 ms preparation delays) did not reach statistical significance (N = 16 subjects, 350 ms: W = 100, *p* = 0.193; 400 ms: W = 88, *p* = 0.215; 450 ms: W = 94, *p* = 0.193; 500 ms: W = 84, *p* = 0.217).

Concerning the early long-latency reflex response (LLR_e_) of the pectoralis, Wilcoxon tests indicated significant goal-directed modulation across both load conditions at all preparatory delays (Fig. 4, middle; N = 16 subjects; Unloaded pectoralis, 250 ms: W = 122, *p* = 0.003, r_rb_ = 0.80; 300 ms: W = 131, *p* < 10^−3^, r_rb_ = 0.93; 350 ms: W = 133, p < 10^−3^, r_rb_ = 0.96; 400 ms: W = 136, *p* < 10^−3^, r_rb_ = 1.00; 450 ms: W = 135, *p* < 10^−3^, r_rb_ = 0.99; 500 ms: W = 135, *p* < 10^–3^, r_rb_ = 0.99; Loaded pectoralis, 250 ms: W = 123, *p* = 0.003, r_rb_ = 0.81; 300 ms: W = 136, *p* < 10^−3^, r_rb_ = 1.00; 350 ms: W = 136, *p* < 10^−3^, r_rb_ = 1.00; 400 ms: W = 133, *p* < 10^−3^, r_rb_ = 0.96; 450 ms: W = 125, *p* = 0.002, r_rb_ = 0.84; 500 ms: W = 127, *p* = 0.001, r_rb_ = 0.87). However, Friedman tests indicated no significant differences in goal-directed tuning of the pectoralis LLR_e_ among the different preparatory delays, under any load condition (Unloaded: χ^2^_4_ = 20.1, *p* = 0.090; Loaded: χ^2^_4_ = 5.4, *p* = 0.708).

The late long-latency reflex responses (LLR_l_) of the pectoralis also showed strong goal-directed tuning across the board, with all relevant Wilcoxon tests yielding W = 136, *p* < 10^−4^ and r_rb_ = 1.00 for all preparatory delays under either load condition (see also Fig. 4, bottom). For the LLR_l_ of the unloaded pectoralis, a Friedman test also indicated a significant impact of preparatory delay (Unloaded: χ^2^_4_ = 27.1, *p* = 0.028; Loaded: χ^2^_4_ = 24.5, *p* = 0.044), with Dunn–Šidák–adjusted post-hoc test indicating a significant difference between the 300 vs. 400 ms delay (*p* = 0.036) when the muscle was unloaded, and a significant difference between the 300 vs 500 ms delays when the muscle was loaded (*p* = 0.036). Note that data on the 250-ms delay were not included in these analyses, as elaborated in the Methods section.

### Goal-directed modulation of the stretch reflex responses – Anterior deltoid

**Fig. 5:**
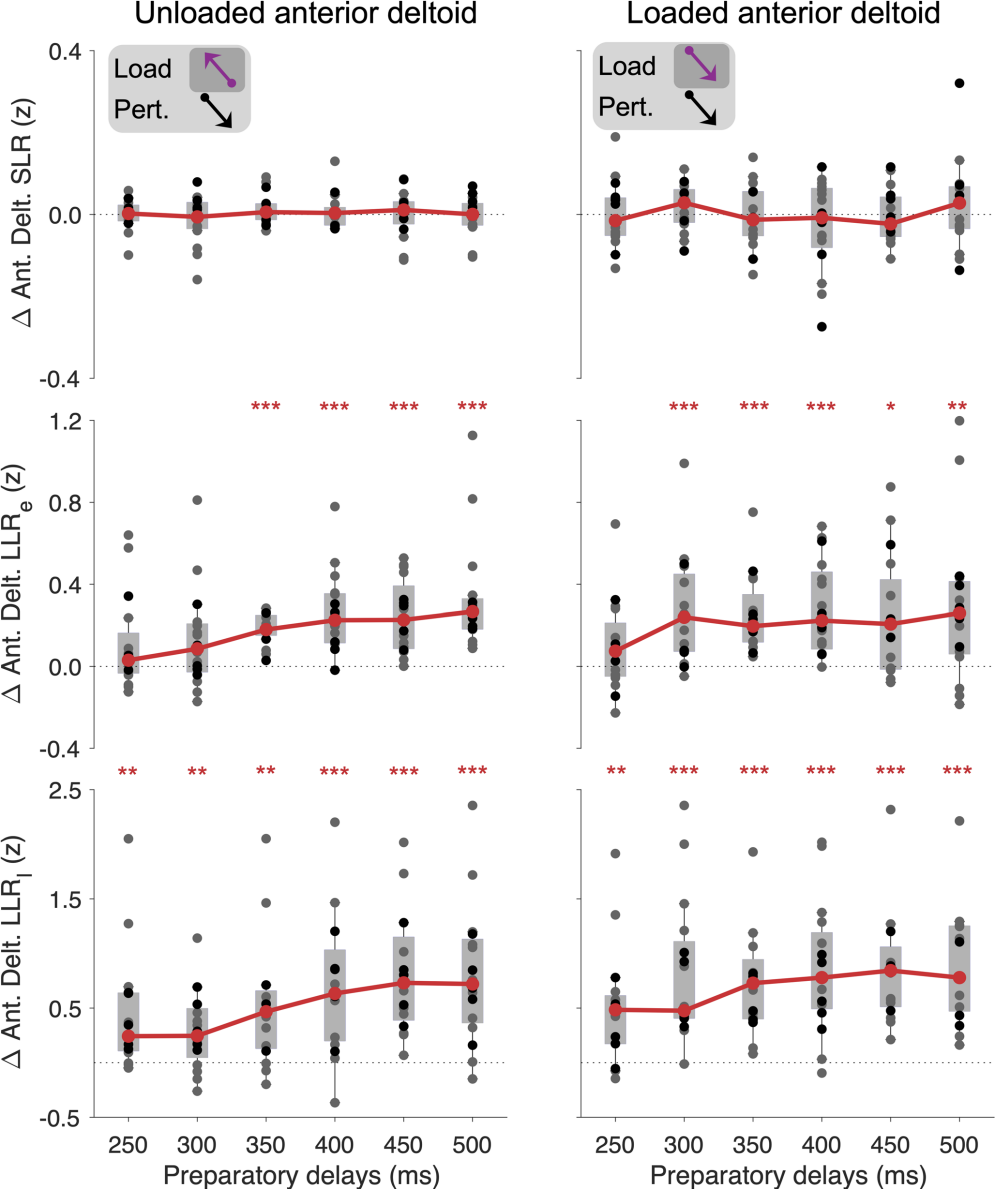
Goal-directed differences in anterior deltoid EMG for the SLR, LLR_e_, and LLR_l_ epochs. Grey circles represent the median single-subject EMG values of the group performing 18 blocks, whereas the black circles represent the values of the group performing 13 blocks. Grey background rectangles represent upper and lower quartiles, and thin vertical lines represent the 95% data range. Solid red lines represent the group-level median values. *p < 0.05, **p < 0.01, ***p < 0.001 denote significant difference from zero

In contrast to the case of its synergist (pectoralis), Wilcoxon tests indicated no significant goal-directed modulation of the anterior deltoid SLR, at any preparatory delay, regardless of load condition (all p > 0.853; Fig. 5, top). However, significant goal-directed tuning of reflex responses was observed at the long latency epochs. Specifically, when the anterior deltoid was unloaded, there was goal-directed tuning of LLR_e_ responses at all preparatory delays except for 250 and 300 ms (N = 16 subjects; 250 ms: W = 92, p = 0.231, r_rb_ = 0.35; 300 ms: W = 105, *p* = 0.069, r_rb_ = 0.54; 350 ms: W = 136, *p* < 10^−4^, r_rb_ = 1.00; 400 ms: W = 135, *p* < 10^−4^, r_rb_ = 0.99; 450 ms: W = 136, *p* < 10^−4^, r_rb_ = 1.00; 500 ms: W = 136, *p* < 10^−4^, r_rb_ = 1.00). The Friedman test indicated a significant impact of preparatory delay on LLR_e_ responses of the unloaded anterior deltoid (χ^2^_4_ = 42.5, *p* = 0.002), with Dunn–Šidák–adjusted post-hoc test indicating a significant difference between the 300 vs. 400 ms delay (*p* = 0.036), and a significant difference between the 300 vs. 500 ms delay (*p* = 0.002; Fig. 5, middle). Inspection of Fig. 5 (middle row) indicates a relatively suppressed goal-directed tuning of the LLR_e_ response at the 300-ms delay. For the loaded anterior deltoid muscle, there was goal-directed tuning of the LLR_e_ at all preparatory delays except 250 ms (N = 16 subjects; 250 ms: W = 98, *p* = 0.141, r_rb_ = 0.44; 300 ms: W = 132, *p* < 10^−3^, r_rb_ = 0.94; 350 ms: W = 136, *p* < 10^−4^, r_rb_ = 1.00; 400 ms: W = 135, *p* < 10^−3^, r_rb_ = 0.99; 450 ms: W = 111, *p* = 0.033, r_rb_ = 0.63; 500 ms: W = 121, *p* = 0.006, r_rb_ = 0.78). For the loaded anterior deltoid, however, the Friedman test indicated no difference in the magnitude of goal-directed LLR_e_ among the 300–500 ms preparatory delays (χ^2^_4_ = 3.9, *p* = 0.818). A direct comparison of anterior deltoid LLR_e_ tuning magnitudes between the loaded and unloaded conditions for the 300 ms preparation delays did reach statistical significance (N = 16 subjects, W = 111, *p* = 0.012).

Similar to the pectoralis, the LLR_l_ response of the anterior deltoid showed goal-directed tuning across the board, with Wilcoxon tests yielding statistical significance for all preparatory delays under both load conditions (N = 16 subjects; Unloaded anterior deltoid, 250 ms: W = 130, *p* < 10^−3^, r_rb_ = 0.91; 300 ms: W = 121, *p* = 0.004, r_rb_ = 0.78; 350 ms: W = 128, *p* < 10^−3^, r_rb_ = 0.88; 400 ms: W = 131, *p* < 10^−3^, r_rb_ = 0.93; 450 ms: W = 136, *p* < 10^−4^, r_rb_ = 1.00; 500 ms: W = 134, *p* < 10^−3^, r_rb_ = 0.97; Loaded anterior deltoid, 250 ms: W = 130, *p* < 10^−3^, r_rb_ = 0.91; 300 ms: W = 135, *p* < 10^−3^, r_rb_ = 0.99; 350 ms: W = 136, *p* < 10^−4^, r_rb_ = 1.00; 400 ms: W = 134, *p* < 10^−3^, r_rb_ = 0.97; 450 ms: W = 136, *p* < 10^−4^, r_rb_ = 1.00; 500 ms: W = 136, *p* < 10^−4^, r_rb_ = 1.00). For the loaded anterior deltoid, the Friedman test indicated no significant differences in goal-directed modulation of the LLR_l_ as a function of preparatory delay (300–500 ms: χ^2^_4_ = 15.6, *p* = 0.181). But there was such an effect when the muscle was unloaded (χ^2^_4_ = 75.9, *p* < 10^−4^), with post-hoc analysis indicating significant differences between 300 vs 400 ms delay (*p* = 0.005), 300 vs 450 ms delay (*p* < 10^−4^), 300 vs 500 ms delay (*p* = 0.012), as well as between 350 vs 450 ms (*p* = 0.002). Inspection of Fig. 5 (bottom row) highlights that the above primarily reflects a diminished goal-directed tuning of the anterior deltoid LLR_l_ when the preparatory delay was 300 ms.

### Goal-directed modulation of stretch reflex responses – Posterior deltoid

**Fig. 6:**
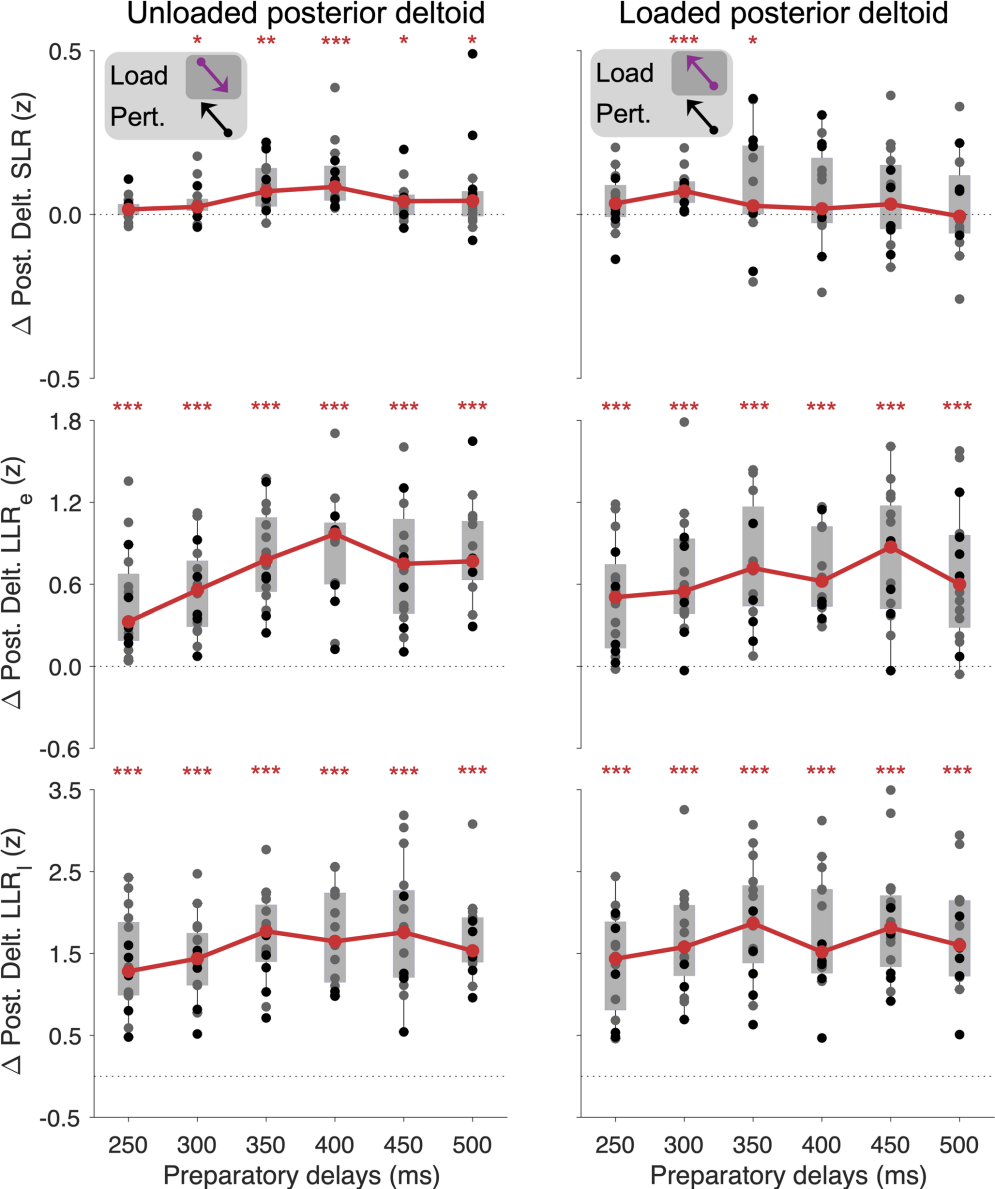
Goal-directed differences in posterior deltoid EMG for the SLR, LLR_e_, and LLR_l_ epochs. Grey circles represent the median single-subject EMG values of the group performing 18 blocks, whereas the black circles represent the values of the group performing 13 blocks. Grey background rectangles represent upper and lower quartiles, and thin vertical lines represent the 95% data range. Solid red lines represent the group-level median values. *p < 0.05, **p < 0.01, ***p < 0.001 denote significant difference from zero

For the SLR response of the unloaded posterior deltoid (Fig. 6, top), Wilcoxon tests indicated significant goal-directed tuning at all preparatory delays except for 250 ms (N = 16 subjects; 250 ms: W = 107, p = 0.067, r_rb_ = 0.57; 300 ms: W = 113, p = 0.044, r_rb_ = 0.66; 350 ms: W = 132, *p* < 10^−3^, r_rb_ = 0.94; 400 ms: W = 136, *p* < 10^−3^, r_rb_ = 1.00; 450 ms: W = 118, *p* = 0.023, r_rb_ = 0.74; 500 ms: W = 110, *p* = 0.050, r_rb_ = 0.62). A Friedman test indicated significant differences in goal-directed SLR responses among the preparatory delays of 300–500 ms (χ^2^_4_ = 38.5, *p* = 0.004) with post-hoc comparisons indicating a significant difference in goal-directed SLR between the 350 and 450 ms delay (*p* = 0.036), as well as the 400 vs 450 ms delay (*p* = 0.036). In contrast to previous findings and the current case of the loaded pectoralis and anterior deltoid muscles, Wilcoxon tests indicated a significant difference in goal-directed tuning of the posterior deltoid SLR, when the muscle was loaded and preparatory delays was 300 ms (W = 136, *p* < 10^−3^, r_rb_ = 1.00) or 350 ms (W = 110, *p* = 0.050, r_rb_ = 0.62). The goal-directed modulation of the SLR at the other preparatory delays was not statistically significant (all *p* > 0.077). A Friedman test indicated no significant and systematic impact of preparatory duration on the loaded posterior deltoid SLR across the 300–500 ms delays (χ^2^_4_ = 11.1, *p* = 0.349). A direct comparison of posterior deltoid SLR tuning magnitudes between the unloaded and loaded conditions for (400 to 500 ms preparation delays) did not reach statistical significance (N = 16 subjects, 400 ms: W = 91, *p* = 0.189; 450 ms: W = 64, *p* = 0.590; 500 ms: W = 92, *p* = 0.189).

The posterior deltoid LLR_e_ (Fig. 6, middle row) showed strong goal-directed tuning across the board, with all relevant Wilcoxon tests yielding W > 134, *p* < 10^−4^ and r_rb_ > 0.98 for all preparatory delays under either load condition. The Friedman test indicated a significant impact of preparatory delay on posterior deltoid LLR_e_ when this muscle was unloaded (χ^2^_4_ = 45.9, *p* = 0.001). The Dunn–Šidák–adjusted post-hoc test indicated a significant difference between the 300 vs. 350 ms delay (*p* = 0.008), between the 300 vs. 400 ms delay (*p* = 0.002) and between the 300 vs. 500 ms delay (*p* = 0.012). Again, visual inspection of Fig. 6 indicates relatively diminished goal-directed tuning of the LLR_e_ when the preparatory delay is 300 ms or shorter. However, when the posterior deltoid was loaded, no significant effect on the LLR_e_ response was found (χ^2^_4_ = 6.6, *p* = 0.618).

The LLR_l_ response of the posterior deltoid (Fig. 6, bottom row) also displayed strong goal-directed tuning across all preparatory delays and load conditions, with all relevant Wilcoxon tests yielding W = 136, *p* < 10^−4^ and r_rb_ = 1.00. As the case for this muscle’s LLR_e_, the Friedman test indicated a significant impact of preparatory delay on the unloaded posterior deltoid LLR_l_ response (χ^2^_4_ = 35.1 and *p* = 0.007), with post-hoc analyses indicating a significant difference between the 300 ms delay vs. 350 and 500 ms delays (*p* = 0.036 and *p* = 0.012, respectively). Again, as the LLR_e_ response, however, the Friedman test indicated no systematic difference in goal-directed tuning of the LLR_l_ when the posterior deltoid was loaded (χ^2^_4_ = 7.2, *p* = 0.575).

In summary, Table 1 presents the results for the pectoralis major, anterior deltoid, and posterior deltoid muscles, regarding the minimum observed preparatory delays associated with goal-directed tuning of responses at the three stretch reflex epochs.

**Table 1:** Shortest preparatory delays where goal-directed tuning of stretch reflexes occurred and facilitation of such tuning with increased preparation

		SLR	LLR_e_	LLR_l_
Shortest preparation for goal-directed tuning				
Pectoralis	Unloaded	350 ms	250 ms	250 ms
	Loaded	n.s.*	250 ms	250 ms
Ant. Delt	Unloaded	n.s	350 ms	250 ms
	Loaded	n.s	300 ms	250 ms
Post. Delt	Unloaded	300 ms	250 ms	250 ms
	Loaded	300 ms	250 ms	250 ms
Enhanced goal-directed tuning with extended preparation (beyond 300 ms)				
Pectoralis	Unloaded	no	no	yes
	Loaded	no	no	yes
Ant. Delt	Unloaded	no	yes	yes
	Loaded	no	no	no
Post. Delt	Unloaded	yes**	yes	yes
	Loaded	no	no	no

n.s, non-significant

*Magnitude of loaded condition is not larger than the unloaded condition, **also evidence of relative decrease at the longest preparation delays

### Onset of the goal-directed modulation of stretch reflex responses

**Fig. 7:**
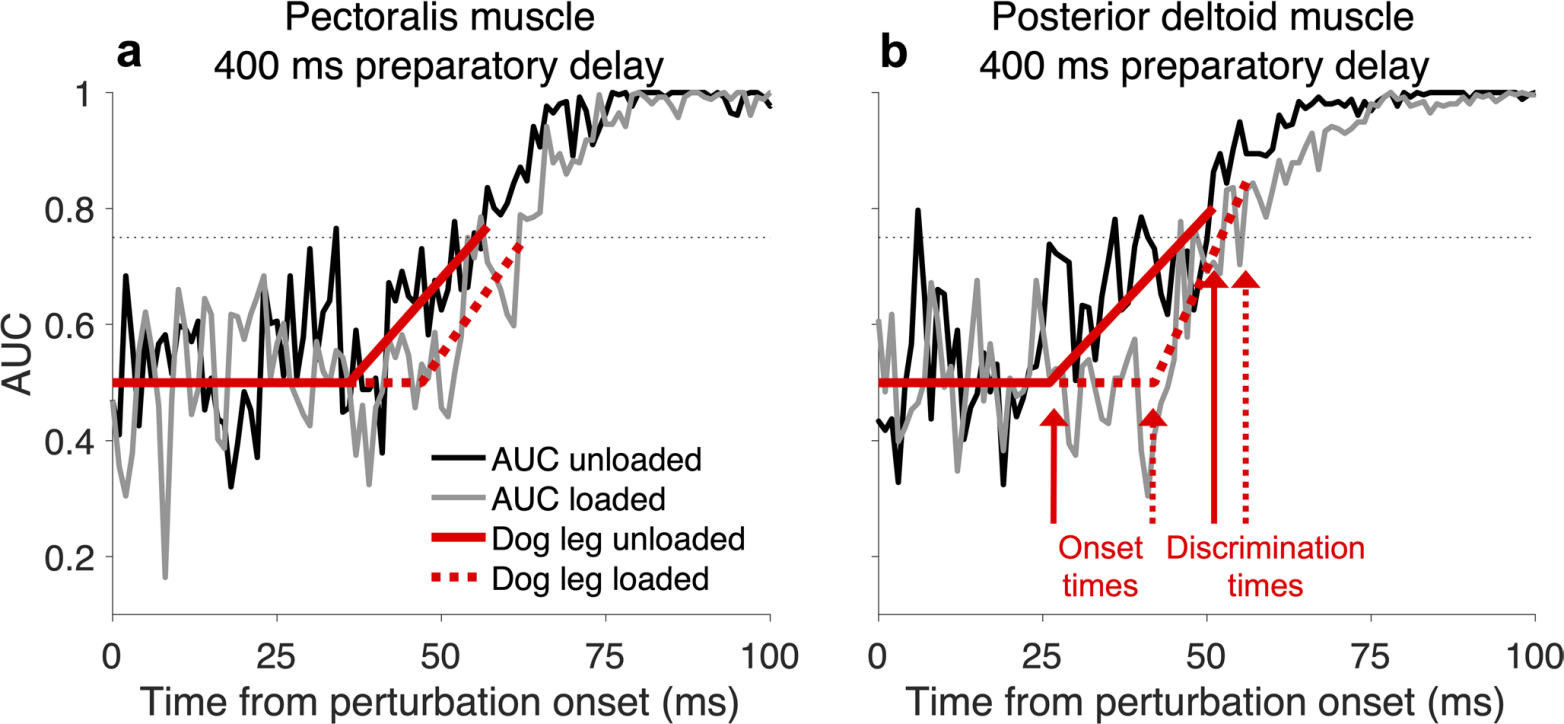
The time onset of SLR modulation with a 400 ms preparatory delay for the pectoralis and posterior deltoid muscles. **a** The area under the curve (AUC) for the ROC values regarding the unloaded (black solid line) and loaded pectoralis (grey solid line) SLR tuning as a function of target direction. The red solid and dotted lines represent the dog leg fits for the unloaded and loaded pectoralis muscle conditions. **b** Same as in (**a**) but for the posterior deltoid muscle. The onset and discrimination times are shown for the unloaded (red solid arrows) and loaded (red dotted arrows) conditions

For the two muscles (pectoralis and posterior deltoid) showing goal-directed tuning of the SLR, we also investigated the onset time of goal-directed discrimination in the unloaded and loaded EMG signals using a sliding ROC analysis (Fig. 7). In addition to onset time, we also investigated the time point at which an ideal observer could discriminate the target position based on the EMG signals. Examples of the AUC values and dog leg fits for the unloaded and loaded (pectoralis and posterior deltoid) muscles with a 400 ms preparatory delay are shown in Fig. 7a–b. From these dog-leg fits, the onset time can be extracted (Fig. 7b), along with the discrimination times for the unloaded and loaded muscles.

**Fig. 8:**
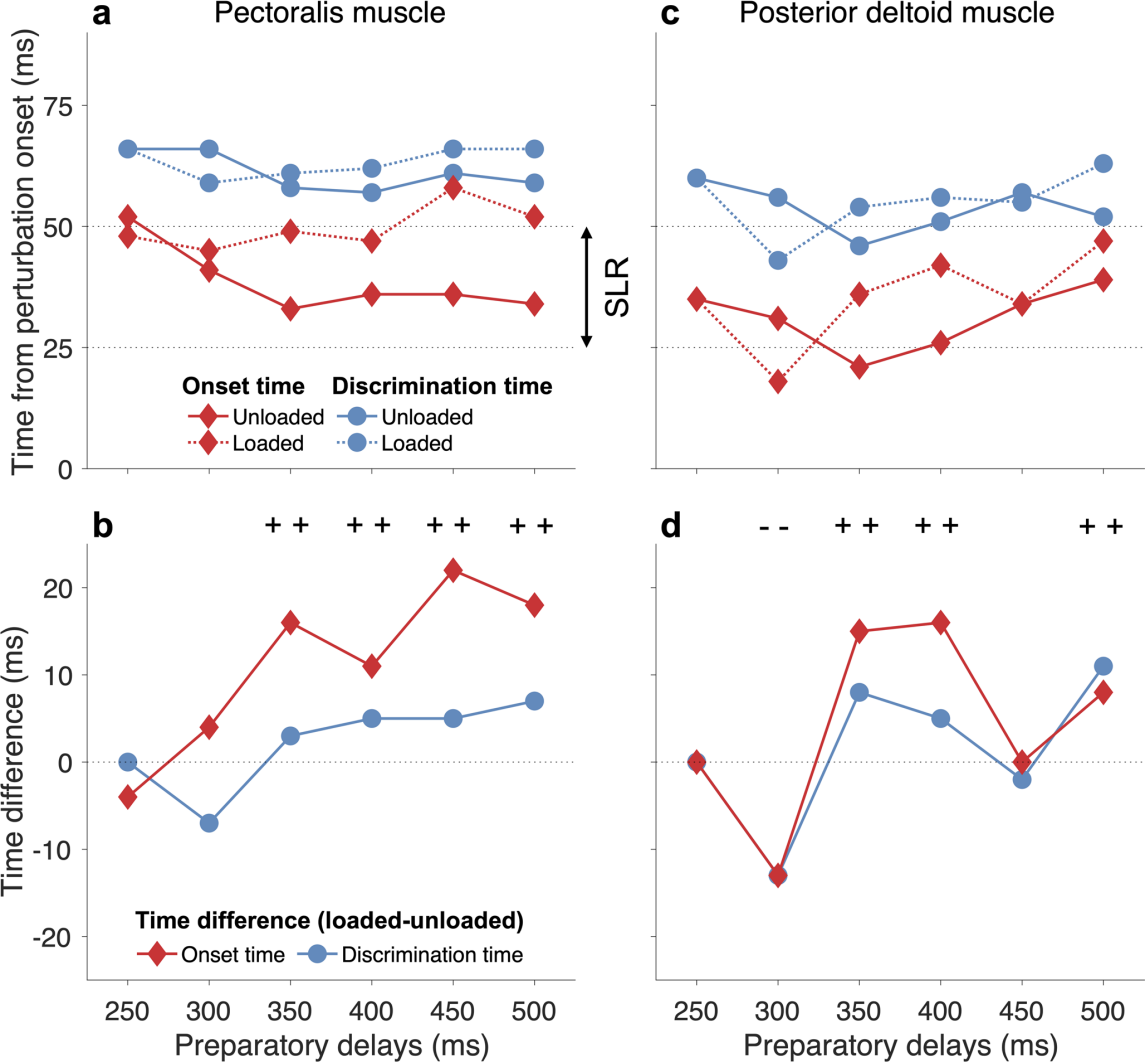
The onset and discrimination times for the pectoralis (**a**–**b**) and posterior deltoid muscles (**c**–**d**). In the top row, the actual times are shown for both the unloaded and loaded muscles. In the bottom row, the time differences (loaded-unloaded) are shown, where ‘ ++ ’ represents when both the onset and discrimination times of the unloaded muscle are shorter than the loaded muscle. In contrast, ‘- -’ represents when both the onset and discrimination times of the unloaded muscle are longer than those of the loaded muscle

For the unloaded pectoralis muscle, the onset and discrimination times for the 250–300 ms preparation delays (41–52 ms and 66 ms) appeared later than the 350–500 ms preparation delays (33–36 ms and 57–61 ms) (Fig. 8a). For the loaded pectoralis, there was no such trend, with onset and discrimination times ranging from 45 to 58 ms and 59 to 66 ms, respectively. By taking into consideration the time difference of the onset and discrimination times between the unloaded and loaded conditions, it is evident that for the 350–500 ms preparation delays, both onset and discrimination times are shorter for the unloaded compared to the loaded pectoralis muscle (denoted ‘ ++  ’ in Fig. 8b). In contrast, for the 250–300 ms preparation delays, either onset or discrimination time is longer. Thus, our findings provide support for the goal-directed tuning observed in the top row of Fig. 4 for the 350–500 ms preparation delays.

In contrast to the pectoralis muscle, the posterior deltoid muscle showed a different trend for the onset and discrimination times (Fig. 8c). For the unloaded muscle, the onset times were 21–39 ms, whereas the discrimination times were 46–60 ms. For the loaded muscle, the onset times were 18–47 ms, whereas the discrimination times were 43–63 ms. The time difference of the onset and discrimination times between the unloaded and loaded conditions revealed that for 300 ms preparatory delay, both onset and discrimination times are longer for the unloaded compared to the loaded pectoralis muscle (denoted ‘- -’ in Fig. 8d). In contrast, for the 350, 400, and 500 ms preparation delays, both onset and discrimination times are shorter for the unloaded compared to the loaded pectoralis muscle (denoted ‘ ++  ’ in Fig. 8d). Thus, these findings for the posterior deltoid muscle provide support for the goal-directed tuning observed in Fig. 6 top row for the 300–500 ms preparation delays.

## Discussion

This study sought to clarify the minimal preparation time required for goal-directed tuning of SLR and LLR responses, and to determine how such tuning progressively evolves with extended preparation. As summarised in Table 1, we found that preparatory delays of 300 ms and 350 ms are sufficient for goal-directed tuning of SLR responses in the posterior deltoid and pectoralis muscles, respectively. Our results also suggest that unloading may facilitate both the earlier emergence and more robust expression of goal-directed SLR tuning (see e.g., top rows of Figs. 4 and 6, and Fig. 8). In contrast to the SLR, goal-directed tuning of LLR responses was already evident at preparation delays of 250 ms, except for the early long-latency reflex of the anterior deltoid muscle. We found no consistent enhancement of goal-directed tuning with extended preparation at the SLR epoch, and that such enhancement was most consistent at the LLR_l_ epoch.

Our results confirm previous findings that preparatory delays of 250 ms are not associated with goal-directed SLR responses (Torell et al. 2023). While pertinent underlying mechanisms may already be at work during preparatory delays of 250 ms or even shorter (Papaioannou and Dimitriou 2021), our study shows that slightly longer preparatory delays (by 50–100 ms) are required to ensure the systematic manifestation of goal-directed tuning of SLR responses, at least as assessed by bipolar surface EMG. Also in line with previous findings, our initial analysis revealed statistically significant goal-directed tuning of the SLR when the homonymous muscle was unloaded (Dimitriou 2018; Torell et al. 2023). It is well known that muscle loading leads to ‘automatic’ gain-scaling of the SLR, regardless of goal or task demands (Pruszynski et al. 2009; Papaioannou and Dimitriou 2021; Torell et al. 2023). That is, perhaps partly via an alpha-gamma coactivation mechanism (Vallbo 1970) or beta-fibre activity (Jami et al. 1982; Emonet-Dénand et al. 1992) in addition to enhanced alpha motor neuron excitability, increased background tonus would shape reflex gains for the purposes of postural maintenance, rather than the facilitation of goal-directed movement per se.

Interestingly, however, the current study reveals that some goal-directed tuning of the posterior deltoid persisted even when this muscle was loaded (Fig. 6, top right), suggesting that muscle loading does not necessarily prevent the manifestation of goal-directed SLR responses. While our initial analysis revealed statistically significant goal-directed tuning of the SLR in the unloaded condition but not in the loaded condition for the most part (for both pectoralis and posterior deltoid muscles), a direct comparison of tuning magnitudes between conditions did not reach significance, consistent with the high variability and low signal-to-noise ratio in stretch reflex responses quantified using EMG. To further probe this difference, we conducted a sliding ROC analysis (Corneil et al. 2004; Pruszynski et al. 2008) to estimate the onset time of goal-directed discrimination in the EMG signals. This analysis revealed that goal-directed tuning emerged (mostly) earlier and more reliably in the unloaded condition compared to the loaded condition (Fig. 8), providing additional support for the idea that unloading facilitates the early expression of preparatory tuning in the sensorimotor system. Moreover, the variability in SLR tuning across muscles is possibly partly due to differences in pre-existing levels of background tone. In addition to recruiting mechanisms (e.g., gamma motor neurons) whose modulation would otherwise serve goal-directed tuning, muscle loading may also obscure the unmasking of goal-directed tuning in the SLR due to corresponding increases in signal-dependent noise (e.g., compare Fig. 6 top left vs. top right).

Nevertheless, as shown for both the posterior deltoid and pectoralis major in our study, unloading a muscle may decrease the tendency of SLRs to respond to any muscle stretch regardless of context, in turn allowing for a more task or goal-directed modulation of reflexes, such as via independent fusimotor control of muscle spindles (Papaioannou and Dimitriou 2021). The additional time required for the goal-directed tuning of SLRs to manifest compared to LLR (> 250 ms) supports the stronger involvement of a slow-evolving mechanism in shaping the former. Indeed, it is known that the slow-conducting fusimotor neurons innervate intrafusal “bag” muscle fibers that are also relatively slow to contract and relax; therefore, effects on spindle firing (i.e., the afferent limb of the stretch reflex) can take several hundred milliseconds to peak or completely disappear following immediate cessation of fusimotor stimulation (Crowe and Matthews 1964).

In contrast to the case of the pectoralis and posterior deltoid muscles, there was no goal-directed tuning of SLR responses in the anterior deltoid muscle. In our task, the anterior deltoid functioned as a synergist of the pectoralis. This, in turn, may imply that, at least after task familiarisation, the sensorimotor system does not apply independent fusimotor control to all synergists potentially engaged in a task but rather to a subset of these. In other words, independent fusimotor control appears highly selective across the muscles that could be similarly engaged as agonists and antagonists in a motor task. Congruent with the idea that feedback controllers are loaded before movement initiation (Ahmadi-Pajouh et al. 2012), we identify the minimum amount of time required by these controllers for manifesting goal-directed modulation of reflex responses, suggesting that feedback controllers likely also engage top-down fusimotor control (Dimitriou 2022).

The top-down control of spinal reflex circuitry has been studied by conditioning the H‐reflex with transcranial magnetic stimulation (TMS), where short preparation delays (200–300 ms) tend to produce strong early cortical inhibition (Lebon et al. 2016). For these short delays, spinal excitability appears to remain unaffected, suggesting that early preparatory inhibition is a top-down process, perhaps partly reflecting a decrease in fusimotor output. In contrast, longer preparation delays (500–900 ms) tend to support more focused and selective inhibition. Considering the findings of Lebon et al. (2016) with our own, it suggests that a window of approximately 300 ms represents the earliest opportunity for implementing selective control across systems, including those shaping the SLR. However, only cortical-dependent pathways (such as those contributing to MEPs and LLRs) continue to fine-tune beyond that point. In contrast, spinal pathways (SLRs and H-reflexes) reach a ceiling in their tuning.

In our study, we show a robust goal-directed modulation of LLR responses, even following the shortest employed preparatory delay of 250 ms. In the context of single-joint movement, Yang and colleagues (Yang et al. 2011) have already shown that LLR gains can be modulated in a task-dependent manner following preparatory delays as short as 70–100 ms. Indeed, ~ 100 ms seems to be the minimum time required for shaping LLR responses via selective CNS processing of proprioceptive signals (Scott 2016). The present study did not examine preparatory delays shorter than 250 ms (see Sect. “Methods” for rationale). Although it is possible that goal-directed modulation of LLR responses can be observed for shorter preparation delays in our paradigm, some deviation from previous findings may be likely due to methodological differences, i.e., our participants prepared and performed multi-joint reaching, rather than single-joint movements, as in the Yang et al. (2011) study. Single-joint movements allow for a more controlled study of the preparatory processes. However, multi-joint movements provide a more naturalistic context for studying how the sensorimotor system coordinates complex actions, such as reach-to-grasp. In other words, it may be the case that the nervous system needs additional time to sufficiently incorporate the goal-directed preparation of multi-joint reaching vs. single-joint movement. Indeed, especially when muscles were unloaded, our results show that the goal-directed tuning of LLR_l_ responses benefited from additional preparation time (Table 1; Figs. 4, 5, 6).

Another interesting phenomenon in the current study was the persistence of ‘false start’ trials, i.e., premature movement initiation. Such trials, although relatively few overall, occurred even though the subjects were instructed to wait for the “Go” signal before and after these ‘false starts’ occurred, suggesting the engagement of specific cognitive mechanisms when the preparatory delay is long enough. The false start trials could occur for several reasons. When a series of alternative preparatory durations is involved in a task, given sufficient preparatory time, participants may explicitly or implicitly attempt to predict when the “Go” cue will occur. This process biases their decision to initiate movement, i.e., anticipating the “Go” cue to come sooner than it does for the longer preparatory delays. Some participants might tend to act more impulsively and, therefore, be more likely to act in anticipation of a “Go” signal. When trying to wait, the CNS (and PNS) prepares for the upcoming movement. The cognitive preparation can sometimes “leak” into premature muscle activation, resulting in a false start (Forgaard et al. 2019). Our findings suggest ≥ 450 ms preparatory delay is sufficient time for this cognitive motor leakage to occur in delayed reach.

In the current study, reflex responses appear relatively inhibited or even absent under certain experimental conditions. This can be attributed to the context-dependent modulation of the stretch reflex. In some situations, the CNS might actively suppress or gate the stretch reflex to allow for controlled movement (Shemmell et al. 2009). If muscle stretch occurs during such higher-order suppression or gating, the resulting contraction might be weaker than expected, potentially being perceived as absent or a minimal response. In the current study, the task strives to be as naturalistic as possible, allowing the subjects to perform the task without being too constrained, which may result in a different recruitment of motor units (Hodson-Tole and Wakeling 2009). Since we used bipolar surface EMG to quantify stretch reflex responses in this naturalistic task, this may partly explain the variability in reflex responses across participants. To address this limitation, the use of multi-channel surface EMG to cover a larger part of the muscle would lead to higher signal-to-noise estimates of the stretch reflex responses, possibly also making it easier to study the data on a trial-by-trial basis and to unmask goal-directed SLR responses. The multi-channel approach also enables the extraction of individual alpha motor neuron spiking activity, where such activity may be reflective of reach plans during preparation (Rungta and Murthy 2023).

It is important to note that in our study we have not adjusted for possible delays associated with the monitor’s refresh rate, software graphics card and response time. Given a 59 Hz refresh rate and a 9 ms response time, our preparation delays may require adjustments of up to 30 ms. However, such an adjustment does not have any qualitative impact on our conclusions, since it corresponds to about half of the size of the increment between two consecutive preparatory delays in our study. Moreover, although our study and its results have been largely framed using computational internal‐model and feedback modulation concepts (Todorov and Jordan 2002; Shadmehr and Krakauer 2008), we recognise that alternative frameworks have been proposed to account for motor control and reflex modulation. Moreover, emerging approaches such as predictive‐coding/active‐inference models (Friston 2010) and bioinspired soft‐robotics paradigms grounded in tissue and muscle mechanics (Capsi-Morales et al. 2021)—provide complementary and physiologically rich accounts of sensorimotor control. A unifying synthesis that bridges feedback‐gain modulation, internal‐model computations, and these emerging frameworks represents an exciting avenue for future studies.

In conclusion, we have assessed how multiple preparatory delays impact the goal-directed modulation of stretch reflex gains of the pectoralis major, anterior deltoid, and posterior deltoid muscles in a multi-joint reaching task. We found that preparatory delays of 300 ms and 350 ms are sufficient for goal-directed tuning of SLR responses in the posterior deltoid and pectoralis muscles, respectively. Our results also suggest that unloading may facilitate both the earlier emergence and more robust expression of goal-directed SLR tuning. We observed goal-directed modulation of LLR responses even after the shortest employed delay of 250 ms. LLR responses were generally robust against background loading conditions, in line with previous findings. Finally, we show that, in contrast to SLR responses, goal-directed tuning of LLR_l_ responses in particular was enhanced when additional preparation time was allowed. Our results help clarify the minimum preparatory time required for goal-directed tuning of stretch reflex gains at the level of the dominant upper limb and characterise the relationship between reflex gains and preparation duration. The inhomogeneous relationship between preparation length and the degree of goal-directed tuning across reflex epochs likely reflects the interplay of multiple feedback mechanisms functioning at different time frames.

## Data Availability

The data that support the findings of this study are available from the corresponding author upon reasonable request.
